# Genome-wide association analysis identifies SNPs predictive of *in vitro* leukemic cell sensitivity to cytarabine in pediatric AML

**DOI:** 10.18632/oncotarget.26163

**Published:** 2018-10-09

**Authors:** Salma A. Bargal, Roya Rafiee, Kristine R. Crews, Huiyun Wu, Xueyuan Cao, Jeffrey E. Rubnitz, Raul C. Ribeiro, James R. Downing, Stanley B. Pounds, Jatinder K. Lamba

**Affiliations:** ^1^ Department of Pharmacotherapy and Translational Research and Center for Pharmacogenomics, College of Pharmacy, University of Florida, Gainesville, FL, USA; ^2^ Department of Pharmaceutical Sciences, St Jude Children's Research Hospital, Memphis, TN, USA; ^3^ Department of Biostatistics, St Jude Children's Research Hospital, Memphis, TN, USA; ^4^ Department of Acute and Tertiary Care, University of Tennessee Health Science Center, Memphis, TN, USA; ^5^ Department of Oncology, St Jude Children's Research Hospital, Memphis, TN, USA; ^6^ Department of Pathology, St Jude Children's Research Hospital, Memphis, TN, USA

**Keywords:** cytarabine, gene expression, GWAS, pediatric acute myeloid leukemia, SNP

## Abstract

Cytarabine has been an integral part of acute myeloid leukemia (AML) chemotherapy for over four decades. However, development of resistance and high rates of relapse is a significant impediment in successfully treating AML. We performed a genome-wide association analysis (GWAS) and identified 113 (83 after adjusting for Linkage Disequilibrium) SNPs associated with *in vitro* cytarabine chemosensitivity of diagnostic leukemic cells from a cohort of 50 pediatric AML patients (p<10^-4^). Further evaluation of diagnostic leukemic cell gene-expression identified 19 SNP-gene pairs with a concordant triad of associations: i)SNP genotype with cytarabine sensitivity (p<0.0001), ii) gene-expression with cytarabine sensitivity (p<0.05), and iii) genotype with gene-expression (p<0.1). Two genes from SNP-gene pairs, rs1376041-*GPR56* and rs75400242-*IGF1R*, were functionally validated by siRNA knockdown in AML cell lines. Consistent with association of rs1376041 and gene-expression in AML patients siRNA mediated knock-down of GPR56 increased cytarabine sensitivity of AML cell lines. Similarly for *IGF1R*, knockdown increased the cytarabine sensitivity of AML cell lines consistent with results in AML patients. Given both *IGF1R* and *GPR56* are promising drug-targets in AML, our results on SNPs driving the expression/function of these genes will not only enhance our understanding of cytarabine resistance but also hold promise in personalizing AML for targeted therapies.

## INTRODUCTION

Acute myeloid leukemia (AML) is the second most common pediatric leukemia with poor outcomes indicated by 5-year survival rates of 50-60%[1, 2]. Cytarabine is one of the most effective chemotherapeutic agents used in AML treatment and has contributed to improved remission rates and overall survival.[3] Although most patients achieve remission after intensive cytarabine-containing induction therapy, a significant proportion of patients experience relapse. Development of resistance to anti-leukemic agents, such as cytarabine, is one of the biggest challenges in achieving successful treatment outcome in AML. Thus, there is a need to better understand the factors that impact the chemosensitivity of leukemic cells. Previous efforts to identify single nucleotide polymorphisms (SNPs) associated with cytarabine resistance in AML have focused on SNPs within candidate pharmacokinetic genes involved in cytarabine metabolism and transport, as well as a few (LCLs) genome-wide efforts studies using of lymphoblastoid cell lines (LCLs)[4–12]. One of the limitations of LCLs used in GWAS studies is that these were derived from healthy individuals that are available through the International HapMap project.[9–12] Given that LCLs do not represent leukemic cells, these cell lines are not the best models to study leukemic cell chemosensitivity to cytarabine. Other efforts have focused on identifying differences in gene expression among AML cell lines with varying sensitivity to cytarabine.[13–19] To the best of our knowledge, no GWAS has so far been performed to identify SNPs that associate with the sensitivity of AML patients’ diagnostic leukemic cells to cytarabine *in vitro*. The difficulty of obtaining bone marrow samples and the *in vitro* cytarabine chemosensitivity in primary AML cells is probably the reason for the lack of such studies, especially in pediatric patients.

## RESULTS

### Patient demographics

The current study included 65 patients enrolled in the multi-center St. Jude AML02 clinical trial with specimens available for determination of *in vitro* leukemic cell cytarabine chemosensitivity. Of these 65 patients, 50 (including 40 white patients) had leukemic cells with *in vitro* cytarabine with IC50 < 5 ng/μL and 15 (including 10 white patients) had leukemic cells that were resistant to cytarabine (IC50 not achieved). The baseline demographics of the patients included in the study are summarized in Table 1. There was no significant difference in age, gender and race between the patients classified as having leukemic cells sensitive or resistant to cytarabine. However, as expected, patients with high-risk group features were more abundant in cytarabine resistant cases. Given that our overall sample size was limited and most (77%) of the participants were genetically white and had a similar proportion of resistant vs. sensitive cases as the whole cohort, we restricted our analysis to the 50 genetically white patients.

**Table 1:** Characteristics of patients included in the present study

Characteristic	All patients^*^ (n=65)			Caucasians (n=50)		
	Resistant (n=15)	Sensitive (n=50)	P-value	Resistant (n=10)	Sensitive (n=40)	P-value
**Age in years (mean±SD)**	11.4 ± 5.2	8.8 ± 6.3	0.159	10.9 ± 4.0	8.6 ± 6.5	0.295
**Female sex**	8 (53.3)	26 (52)	0.928	5 (50)	21 (52.5)	0.887
**Race**			0.282			-
**Caucasian**	10 (66.7)	40 (80)		-	-	
**Black**	5 (33.3)	10 (20)		-	-	
**Initial risk group**			0.002			0.002
**Low**	2 (13.3)	17 (34)		0 (0)	14 (35)	
**Standard**	3 (20)	24 (48)		3 (30)	20 (50)	
**High**	10 (66.7)	9 (18)		7 (70)	6 (15)	

Values are presented as number (percentage) unless otherwise noted.

^*^ All patients comprise of 50 Caucasians and 15 Blacks.

### Genome-wide association analysis

**Figure 1:**
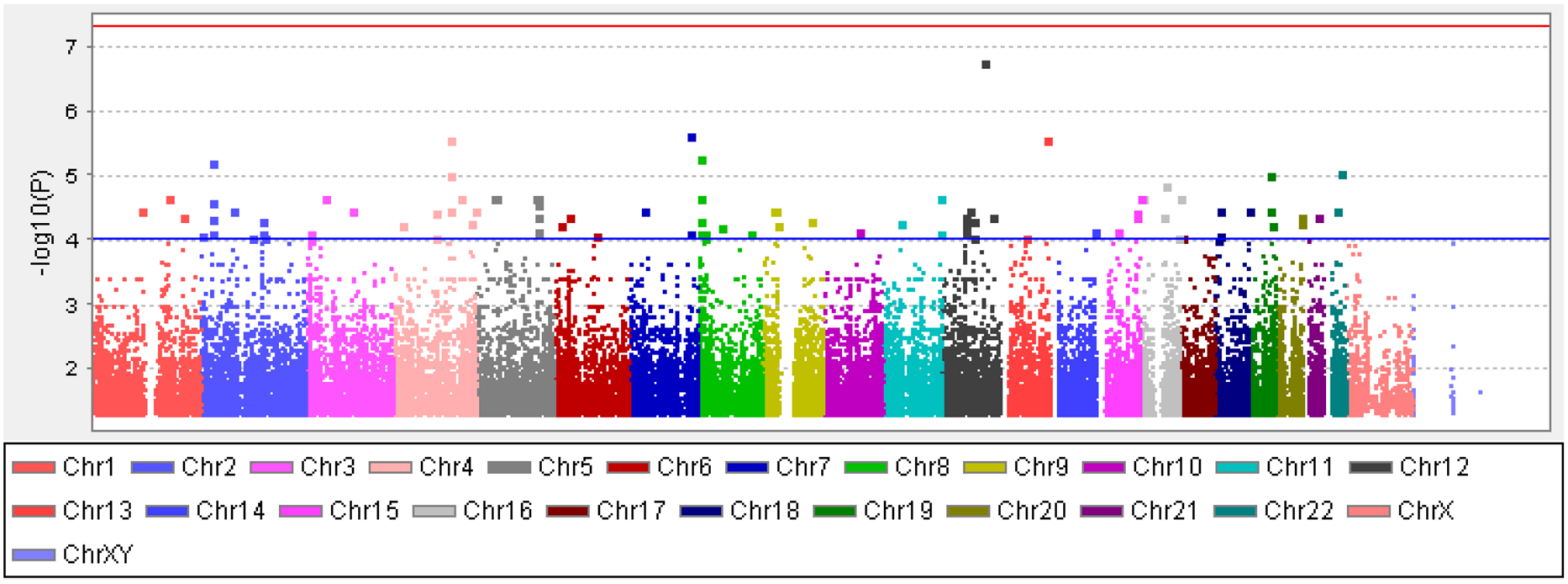
The Manhattan plot showing results of the genome-wide SNP association analysis with *in vitro* cytarabine IC50 of diagnostic leukemic cells within white pediatric AML patients The y-axis is – log10 the association p-value for the SNPs and the x-axis shows the chromosomal locations of the SNPs. The red line is the genome-wide significance threshold (P=5x10^-8^) and the blue line is the suggestive threshold (P=1x10^-4^).

After rigorous quality control (QC) as described in the methods below, we evaluated 1,317,106 variants in 50 white patients. With this limited sample size, none of the SNPs reached genome-wide significance at the Bonferroni threshold for the familywise error rate (P<5 × 10^-8^; Figure 1). Nevertheless, as our study likely has the largest feasible sample size in this rare disease setting, we selected 113 SNPs with p < 0.0001 to evaluate for further scientific evidence of involvement in cytarabine response (Table 2).

**Table 2:** List of 113 SNPs with suggestive level of association (P<1.0x10^-4^) with *in vitro* cytarabine chemo-sensitivity of diagnostic leukemic cells from AML patients enrolled on multicenter St. Jude AML02 clinical trial

SNP	Chr	Location relative to nearest gene	Gene Name	P-value	Direction of association with minor allele
rs17030128	1	37kb 5' of CTTNBP2NL	CTTNBP2 N-terminal like	0.00004	Resistance
rs78755594	1	509kb 5' of LOC642587	MIR205 host gene	0.00004	Resistance
rs12730413	1	Within TNFSF4	Tumor necrosis factor superfamily, member 4	0.00002	Resistance
rs11123318	2	Within DPP10	Dipeptidyl peptidase like 10	0.00009	Resistance
rs2384234	2	Within DTNB	Dystrobrevin beta	0.00001	Resistance
rs12472283	2	Within DTNB	Dystrobrevin beta	0.00003	Resistance
rs13025966	2	Within DTNB	Dystrobrevin beta	0.00003	Resistance
rs17745923	2	Within DTNB	Dystrobrevin beta	0.00003	Resistance
rs34104763	2	Within DTNB	Dystrobrevin beta	0.00005	Resistance
rs34736805	2	Within DTNB	Dystrobrevin beta	0.00005	Resistance
rs35921400	2	Within DTNB	Dystrobrevin beta	0.00008	Resistance
rs1869686	2	16kb 5' of HNMT	Histamine N-methyltransferase	0.00008	Resistance
rs2241028	2	Within HTRA2	HtrA serine peptidase 2	0.00004	Resistance
rs1437352	2	Within LRP1B	Low density lipoprotein receptor related protein 1B	0.00005	Sensitivity
rs16847711	2	Within LRP1B	Low density lipoprotein receptor related protein 1B	0.00009	Resistance
rs7565278	2	5' of TGFA and 26kb 3' of ADD2	Transforming growth factor alpha and adducin 2	0.00004	Resistance
rs12714411	2	60kb 3' of TMEM18	Transmembrane protein 18	0.00008	Resistance
rs16850901	3	215kb 5' of ALCAM	Activated leukocyte cell adhesion molecule	0.00004	Resistance
rs11719029	3	17kb 5' of CX3CR1	C-X3-C motif chemokine receptor 1	0.00002	Resistance
rs11927011	3	723kb 5' of MIR4790	MicroRNA 4790	0.00008	Resistance
rs6773395	3	725kb 5' of MIR4790	MicroRNA 4790	0.00008	Resistance
rs9835716	3	735kb 5' of MIR4790	MicroRNA 4790	0.00010	Resistance
rs11934226	4	970kb 3' of C4orf33	Chromosome 4 open reading frame 33	0.00000	Resistance
rs7664923	4	958kb 3' of C4orf33	Chromosome 4 open reading frame 33	0.00000	Resistance
rs111261849	4	973kb 3' of C4orf33	Chromosome 4 open reading frame 33	0.00001	Resistance
rs62309493	4	460kb 3' of C4orf33	Chromosome 4 open reading frame 33	0.00004	Resistance
rs4253418	4	Within F11	Coagulation factor XI	0.00004	Resistance
rs79827604	4	9.6kb 3' of PRSS48	Protease, serine 48	0.00002	Resistance
rs614158	4	4.1kb 5' of SLIT2	Slit guidance ligand 2	0.00006	Resistance
rs640501	4	5.4kb 5' of SLIT2	Slit guidance ligand 2	0.00006	Resistance
rs9991282	4	Within UNC5C	Unc-5 netrin receptor C	0.00004	Resistance
rs73838827	4	Within UNC5C	Unc-5 netrin receptor C	0.00009	Resistance
rs2062416	4	60kb 3' of VEGFC	Vascular endothelial growth factor C	0.00005	Resistance
rs62370525	5	232kb 3' of FBXO4	F-box protein 4	0.00002	Resistance
rs34115593	5	Within FGF1	Fibroblast growth factor 1	0.00002	Resistance
rs71587226	5	Within FGF1	Fibroblast growth factor 1	0.00003	Resistance
rs6884797	5	Within FGF1	Fibroblast growth factor 1	0.00004	Resistance
rs17574650	5	Within GHR	Growth hormone receptor	0.00002	Resistance
rs9324859	5	3.1kb 5' of LOC729080	Glycine cleavage system protein H (aminomethyl carrier) pseudogene	0.00007	Resistance
rs17797667	5	25kb 3' of NPY6R	neuropeptide Y receptor Y6 (pseudogene)	0.00002	Resistance
rs4645358	5	2.1kb 5' of PCDH1	protocadherin 1	0.00007	Resistance
rs72923772	6	277kb 5' of MIR4643	microRNA 4643	0.00008	Resistance
rs9368620	6	NEDD9	neural precursor cell expressed	0.00006	Resistance
rs356973	6	ZNRD1-AS1	Zinc Ribbon Domain Containing 1 Antisense, Pseudogene	0.00004	Resistance
rs17798136	7	39kb 3' of BBS9	Bardet-Biedl syndrome 9	0.00004	Resistance
rs2174362	7	5.9kb 5' of ZC3HAV1	zinc finger CCCH-type containing, antiviral 1	0.00000	Resistance
rs76651618	7	10kb 5' of ZC3HAV1	zinc finger CCCH-type containing, antiviral 1	0.00000	Resistance
rs59520763	7	2.3kb 5' of ZC3HAV1	zinc finger CCCH-type containing, antiviral 1	0.00008	Resistance
rs270066	8	CSMD1	CUB and Sushi multiple domains 1	0.00001	Resistance
rs4242513	8	CSMD1	CUB and Sushi multiple domains 1	0.00002	Resistance
rs4493928	8	CSMD1	CUB and Sushi multiple domains 1	0.00002	Resistance
rs55824821	8	CSMD1	CUB and Sushi multiple domains 1	0.00005	Resistance
rs12542120	8	CSMD1	CUB and Sushi multiple domains 1	0.00008	Resistance
rs897266	8	DLC1	DLC1 Rho GTPase activating protein	0.00008	Sensitivity
rs7018361	8	DLC1	DLC1 Rho GTPase activating protein	0.00009	Sensitivity
rs16939055	8	6.2kb 3' of SNAI2	snail family transcriptional repressor 2	0.00006	Resistance
rs13270346	8	TRPS1	transcriptional repressor GATA binding 1	0.00008	Resistance
rs199615944	9	1.8kb 5' of C9orf144	Family With Sequence Similarity 205 Member B, Pseudogene	0.00006	Resistance
rs72717694	9	45kb 5' of MIR873	microRNA 873	0.00004	Resistance
rs16936272	9	506kb 3' of TMEM38B	transmembrane protein 38B	0.00005	Resistance
rs6477500	9	502kb 3' of TMEM38B	transmembrane protein 38B	0.00005	Resistance
rs77798124	9	500kb 3' of TMEM38B	transmembrane protein 38B	0.00005	Resistance
rs7854098	9	502kb 3' of TMEM38B	transmembrane protein 38B	0.00005	Resistance
rs10966448	9	909kb 3' of TUSC1	tumor suppressor candidate 1	0.00004	Resistance
rs884889	10	1.6kb 3' of ZMIZ1	Zinc Finger MIZ-Type Containing 1	0.00007	Resistance
rs12364729	11	30kb 5' of LOC100507205	uncharacterized LOC100507205	0.00005	Resistance
rs77600532	11	OPCML	opioid binding protein/cell adhesion molecule like	0.00002	Resistance
rs545375	11	OPCML	opioid binding protein/cell adhesion molecule like	0.00008	Resistance
rs303825	12	1.7kb 3' of FIGNL2	fidgetin like 2	0.00006	Resistance
rs11067996	12	72kb 5' of MED13L	Mediator Complex Subunit 13 Like	0.00004	Resistance
rs721947	12	93kb 3' of NEDD1	neural precursor cell expressed	0.00000	Resistance
rs303815	12	SCN8A	sodium voltage-gated channel alpha subunit 8	0.00004	Resistance
rs303817	12	SCN8A	sodium voltage-gated channel alpha subunit 8	0.00004	Resistance
rs60637	12	SCN8A	sodium voltage-gated channel alpha subunit 8	0.00004	Resistance
rs747283	12	SCN8A	sodium voltage-gated channel alpha subunit 8	0.00007	Resistance
rs1905248	12	SCN8A	sodium voltage-gated channel alpha subunit 8	0.00008	Resistance
rs76411845	12	TBC1D30	TBC1 domain family member 30	0.00004	Resistance
rs1112973	12	633kb 3' of TRHDE	thyrotropin releasing hormone degrading enzyme	0.00005	Sensitivity
rs7301109	12	635kb 3' of TRHDE	thyrotropin releasing hormone degrading enzyme	0.00009	Sensitivity
rs7996008	13	63kb 3' of PCDH17	protocadherin 17	0.00009	Resistance
rs72632692	13	1Mb 5' of SLC10A2	solute carrier family 10 member 2	0.00000	Resistance
rs58540528	14	33kb 3' of DLK1	delta like non-canonical Notch ligand 1	0.00007	Resistance
rs75400242	15	147kb 3' of FAM169B/360 kb upstream of IGF1R	Insulin like growth factor receptor 1	0.00002	Resistance
rs1974961	15	SHC4	SHC adaptor protein 4	0.00007	Resistance
rs62011577	15	SLCO3A1	solute carrier organic anion transporter family member 3A1	0.00004	Resistance
rs7182304	15	SLCO3A1	solute carrier organic anion transporter family member 3A2	0.00004	Resistance
rs9889220	16	3.2kb 3' of ADAD2	adenosine deaminase domain containing 2	0.00009	Resistance
rs1376041	16	GPR56	G Protein-Coupled Receptor 56	0.00001	Resistance
rs8051448	16	JPH3	junctophilin 3	0.00002	Resistance
rs9922685	16	SRL	sarcalumenin	0.00002	Resistance
rs7203855	16	72kb 5' of ZNF423	zinc finger protein 423	0.00004	Resistance
rs9911336	17	SLC43A2	solute carrier family 43 member 2	0.00009	Resistance
rs9967269	18	DLGAP1	DLG associated protein 1	0.00010	Sensitivity
rs34381217	18	852kb 3' of GALR1	galanin receptor 1	0.00004	Resistance
rs4796870	18	169kb 3' of VAPA	VAMP associated protein A	0.00004	Resistance
rs9957328	18	163kb 3' of VAPA	VAMP associated protein A	0.00008	Resistance
rs2968180	19	BCAM	basal cell adhesion molecule	0.00001	Resistance
rs7249750	19	BCAM	basal cell adhesion molecule	0.00004	Resistance
rs4801739	19	BSPH1	binder of sperm protein log 1	0.00006	Resistance
rs10408993	19	1.8kb 3' of CBLC	Cbl proto-oncogene C	0.00004	Resistance
rs78253907	19	CBLC	Cbl proto-oncogene C	0.00004	Resistance
rs4925251	20	CDH4	cadherin 4	0.00004	Resistance
rs6142655	20	CDH4	cadherin 4	0.00004	Resistance
rs6142792	20	CDH4	cadherin 4	0.00004	Resistance
rs62201785	20	CDH4	cadherin 4	0.00004	Resistance
rs67283024	20	CDH4	cadherin 4	0.00004	Resistance
rs4925252	20	CDH4	cadherin 4	0.00005	Resistance
rs34517760	21	47kb 5' of SOD1	superoxide dismutase 1, soluble	0.00004	Resistance
rs56837868	22	495kb 3' of MN1	Meningioma 1 proto-oncogene (Transcriptional Regulator)	0.00004	Resistance
rs2072712	22	NCF4	neutrophil cytosolic factor 4	0.00001	Resistance
rs34072125	22	1.7kb 3' of NCF4	neutrophil cytosolic factor 4	0.00001	Resistance
rs5995360	22	NCF4	neutrophil cytosolic factor 4	0.00001	Resistance
rs6000462	22	287bp 3' of NCF4	neutrophil cytosolic factor 4	0.00001	Resistance

Abreviations: Chr: chromosome; SNP: Single nucleotide polymorphism.

The top GWAS SNP associated with cytarabine resistance is rs721947 (P=1.77x10^-7^). It is located on chromosome 12 downstream of *NEDD1* and co-localized within a long intergenic non-coding RNA (LincRNA) region. A few other potentially interesting SNPs localized within or close to the genes of potential relevance in myeloid malignancies. A synonymous SNP, rs1376041 G>A (P=1.42x10^-5^), is located within a conserved region close to exon/intron splice junction within the G protein-coupled receptor 56 (*GPR56*) gene. Presence of the minor allele A for rs1376041 was associated with cytarabine resistance. Our results on this SNP are very exciting given that *GPR56* contributes to AML development and its expression has been associated with inferior outcome in AML.[18, 20] rs75400242 G>A (P=2.1x10^-5^) SNP is located in a region upstream of *IGF1R* (Insulin like growth factor receptor) a gene expressed in human leukemia cell lines with pathological significance in AML [21–23]. *IGF1R* is among one of the significantly phosphorylated receptor tyrosine kinase in AML that has been associated with activation of PI3K/AKT signaling and thus cell growth and survival in AML [23].

rs56837868 G>A (P=3.56x10^-5^) is located in a LincRNA ENSG00000233574 and is roughly 495 kb downstream of the transcriptional co-activator Meningioma1 (*MN1*) proto-oncogene. *MN1* overexpression has been reported to be an important step in the development of inv(16) AML, and is predictive of poor outcome in AML patients with normal cytogenetics.[24, 25] An intronic SNP, rs545375 A>G (P=7.67x10^-5^), in opioid binding protein/cell adhesion molecule like (*OPCML*) occurs in high LD (r^2^=0.93, D’=1) with rs540923, which is an eQTL for Homeobox A10 (*HOXA10*), a well-known myeloid leukemia gene. rs7565278 is present around 26kb 3’ of the adducin 2 beta (*ADD2*) gene and approximately 85kb 5’ of the transforming growth factor alpha (*TGFA*) gene, which is expressed in neoplastic myeloblasts.

Several SNPs were located in or near cell adhesion genes (*ALCAM, BCAM, CDH4, NEDD9, OCML, PCDH1* and *PCDH17*) and genes involved death receptor signaling pathways (*HTRA2* and *ZC3HAV1a* PARP family member). SNPs with in or close to other biologically interesting genes included: *CXC3R1, a* chemokine receptor which is expressed at higher levels in AML [26]; *DLK1*, a member of the NOTCH signally pathway with a potentially oncogenic role in myeloid dysplastic syndrome [27], a disease that frequently progresses into AML; *VEGFC* (vascular endothelial growth factor C), for which higher expression has been previously associated with chemo-resistance and adverse prognosis in AML [28].

### Expression quantitative trait loci (eQTL) analysis for differentially expressed genes

**Figure 2:**
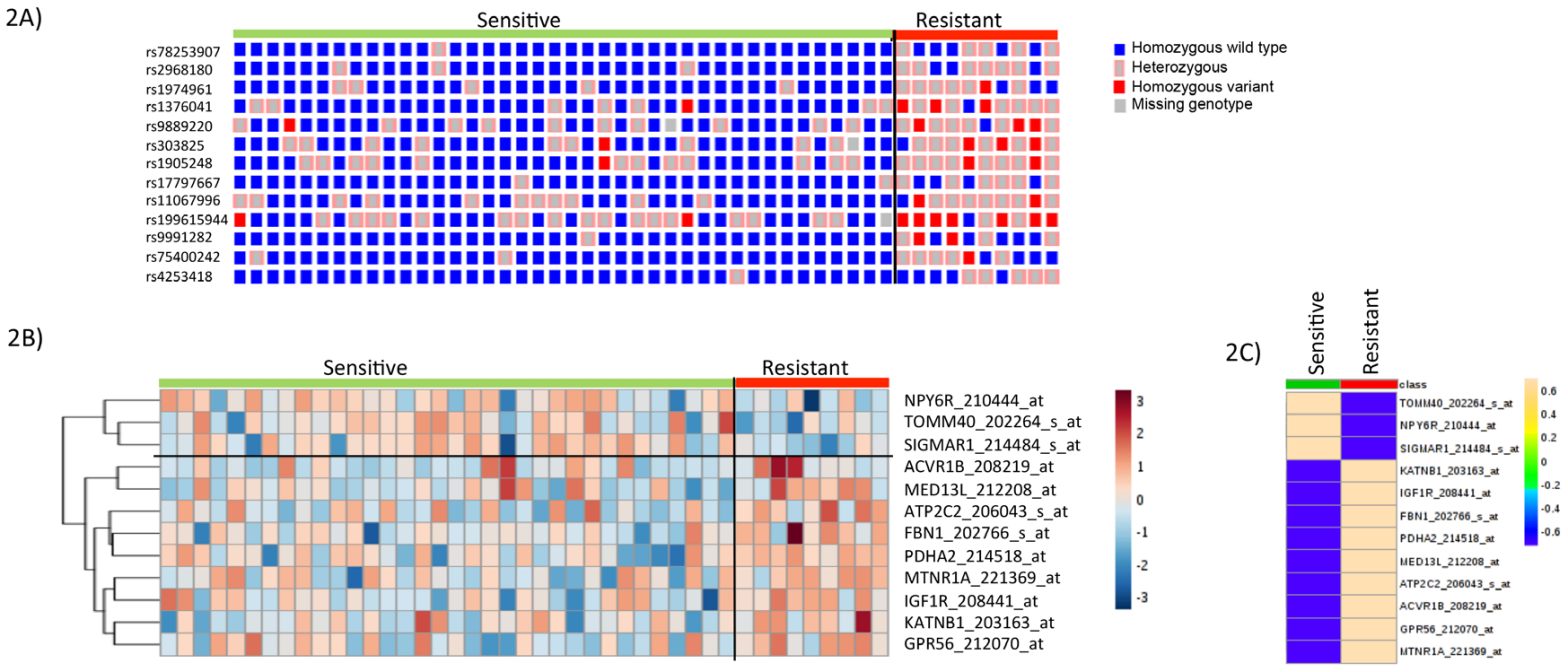
SNP-genotype and gene-expression heat-map in cytarabine sensitive and resistant cases **(A)** Genotype heat-map for 13 unique SNPs between cytarabine sensitive and resistant cases. **(B)** Gene-expression heat-map of 12 genes showing expression differences between cytarabine sensitive and resistant cases. **(C)** Average gene-expression of 12 genes between cytarabine sensitive and resistant cases.

We then evaluated the association of the expression of the genes using 578 unique microarray probe sets located within 500kb of the 113 SNPs identified in the GWAS analysis above. Thirty-eight probes representing 38 unique genes showed significant differential expression between the 10 cytarabine-resistant cases and the 40 cytarabine-sensitive cases (p < 0.05; Supplementary Table 1). Next, we determined cis-eQTL associations between expression levels of the 38 genes with differential expression between sensitive and resistant cell lines and the 113 identified SNPs. We identified 19 SNP-gene pairs among 13 unique SNPs and 12 unique genes with a concordant triad of associations (Table 3): SNP genotype with cytarabine sensitivity (p < 0.0001), mRNA expression with cytarabine sensitivity (p < 0.05), and SNP genotype with mRNA expression (p < 0.1). Five SNP-gene pairs were informatively redundant due to linkage among SNPs. Figures 2A-2C provides heat-maps illustrating the genotype distribution of the 13 SNPs between cytarabine sensitive and resistant leukemia cells as well expression levels of the 12 genes between cytarabine sensitive and resistant leukemia cells.

**Table 3:** List of candidate SNP-gene pairs that passed all the three criteria -i) SNP associated with cytarabine *in vitro* sensitivity ii) gene within 500+/- of SNP was differentially expressed in resistant vs. sensitive cases and iii) SNP is associated with gene expression

SNP	Probe ID	Gene Symbol	SNP-gene expression (*cis*-eQTL) P-value	Minor Allele association direction with gene-expression	Gene-expression vs. cytarabine *in vitro* sensitivity (P-value)	Gene-expression association direction in Resistant Cells	SNP vs. cytarabine *in vitro* Sensitivity P-value	Minor allele association direction with cytarabine *in vitro* sensitivity
rs78253907	202264_s_at	*TOMM40*	0.023	Low	0.022	Low	0.0000356	Resistant
rs2968180	202264_s_at	*TOMM40*	0.010	Low	0.022	Low	0.0000099	Resistant
rs1974961	202766_s_at	*FBN1*	0.046	High	0.006	High	0.0000749	Resistant
rs1376041	203163_at	*KATNB1*	0.049	High	0.046	High	0.0000142	Resistant
rs9889220	206043_s_at	*ATP2C2*	0.040	High	0.034	High	0.0000899	Resistant
rs303825	208219_at	*ACVR1B*	0.019	High	0.029	High	0.0000551	Resistant
rs1905248	208219_at	*ACVR1B*	0.009	High	0.029	High	0.0000767	Resistant
rs17797667	210444_at	*NPY6R*	0.016	Low	0.034	Low	0.000022	Resistant
rs1376041	212070_at	*GPR56*	0.022	High	0.036	High	0.0000142	Resistant
rs11067996	212208_at	*MED13L*	0.033	High	0.014	High	0.0000442	Resistant
rs199615944	214484_s_at	*SIGMAR1*	0.040	Low	0.006	Low	0.0000558	Resistant
rs9991282	214518_at	*PDHA2*	0.023	High	0.003	High	0.0000374	Resistant
rs75400242	rs208441_at	*IGF1R*	0.062	High	0.031	High	0.0000219	Resistant
rs4253418	221369_at	*MTNR1A*	0.031	High	0.026	High	0.0000356	Resistant

rs78253907 within TOMM40 occurs in LD (r2=1) with rs10408993 and rs7249750rs303825 in ACVR1B occurs in LD (r2 =1) with rs303815, rs60637 and rs303817.

Guided by the results above and existing evidence from literature on genes with relevance in AML, we further selected *GPR56* and *IGF1R* for further *in vitro* validation of the impact on cytarabine resistance in AML cell lines.

### rs1376041-*GPR56*

**Figure 3:**
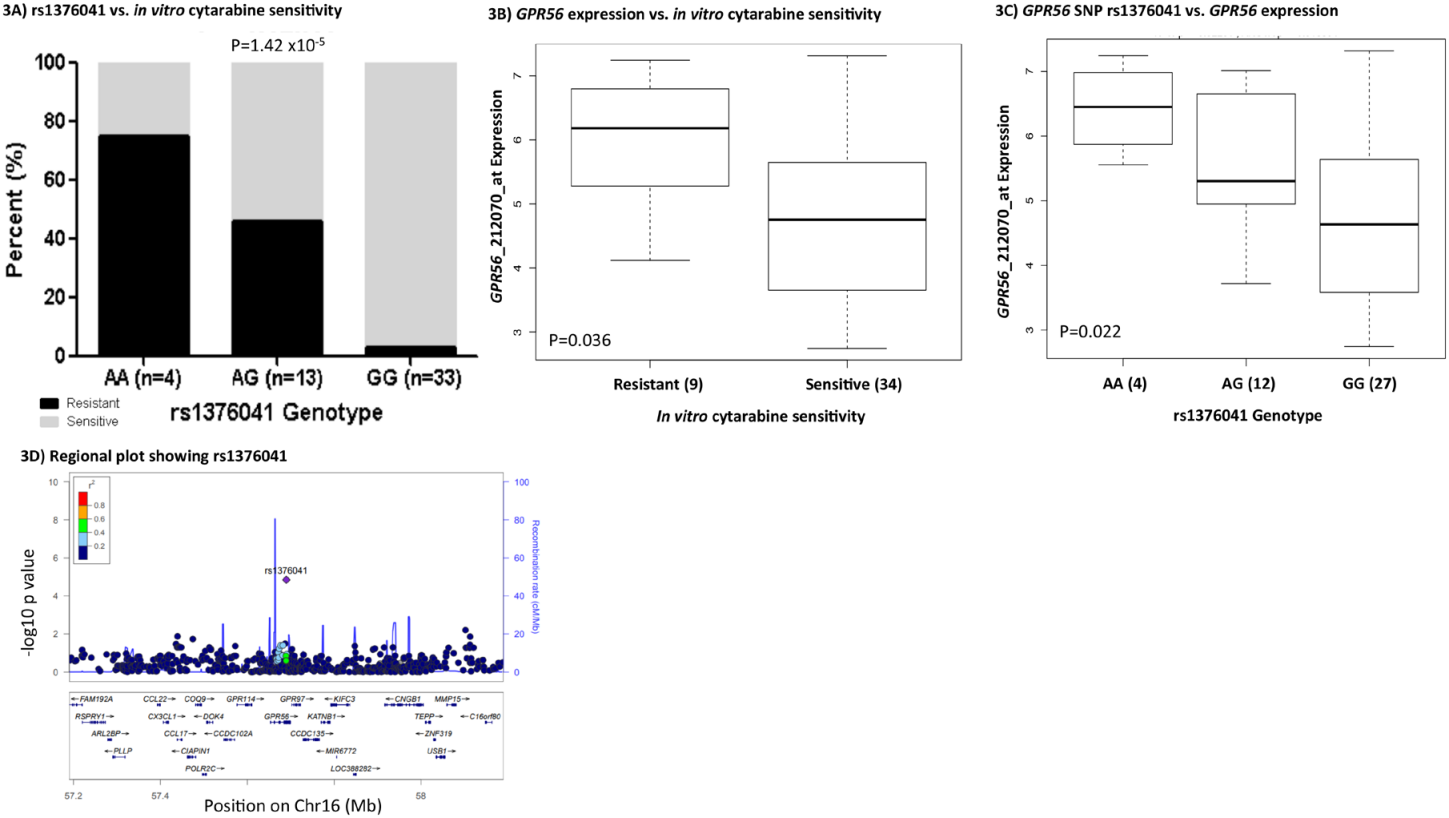
*GPR56* and rs1376041 **(A)** Bar-plot showing the distribution of cytarabine sensitive and resistant cases within genotype groups for rs1376041. **(B)** Boxplot showing the association of leukemic cell *GPR56* gene expression levels between cytarabine sensitive and resistant cases. **(C)** Boxplot demonstrating association of rs1376041 genotypes with *GPR56* gene expression. In boxplots, the y-axis represents natural logarithm of gene expression and the x-axis shows the sensitive/resistant groups or genotype groups. The horizontal line inside the box represents the median value of each group, the lower hinge of the box represents the 25th percentile, the upper hinge represents the 75th percentile and the lower and upper whiskers here display 1.5 times the interquartile range. The outliers are defined as data points that fall outside of the first and third quartiles by more than 1.5 times the interquartile range. **(D)** Regional plot showing -log10 p-value of the association of SNPs with cytarabine *in vitro* sensitivity on the y-axis and genes on chromosome 16 within +/- 500kb from rs1376041 on the x-axis. As denoted by the legend with colors indicating linkage disequilibrium, rs1376041 was not in linkage disequilibrium with other SNPs within the region.

**Figure 4:**
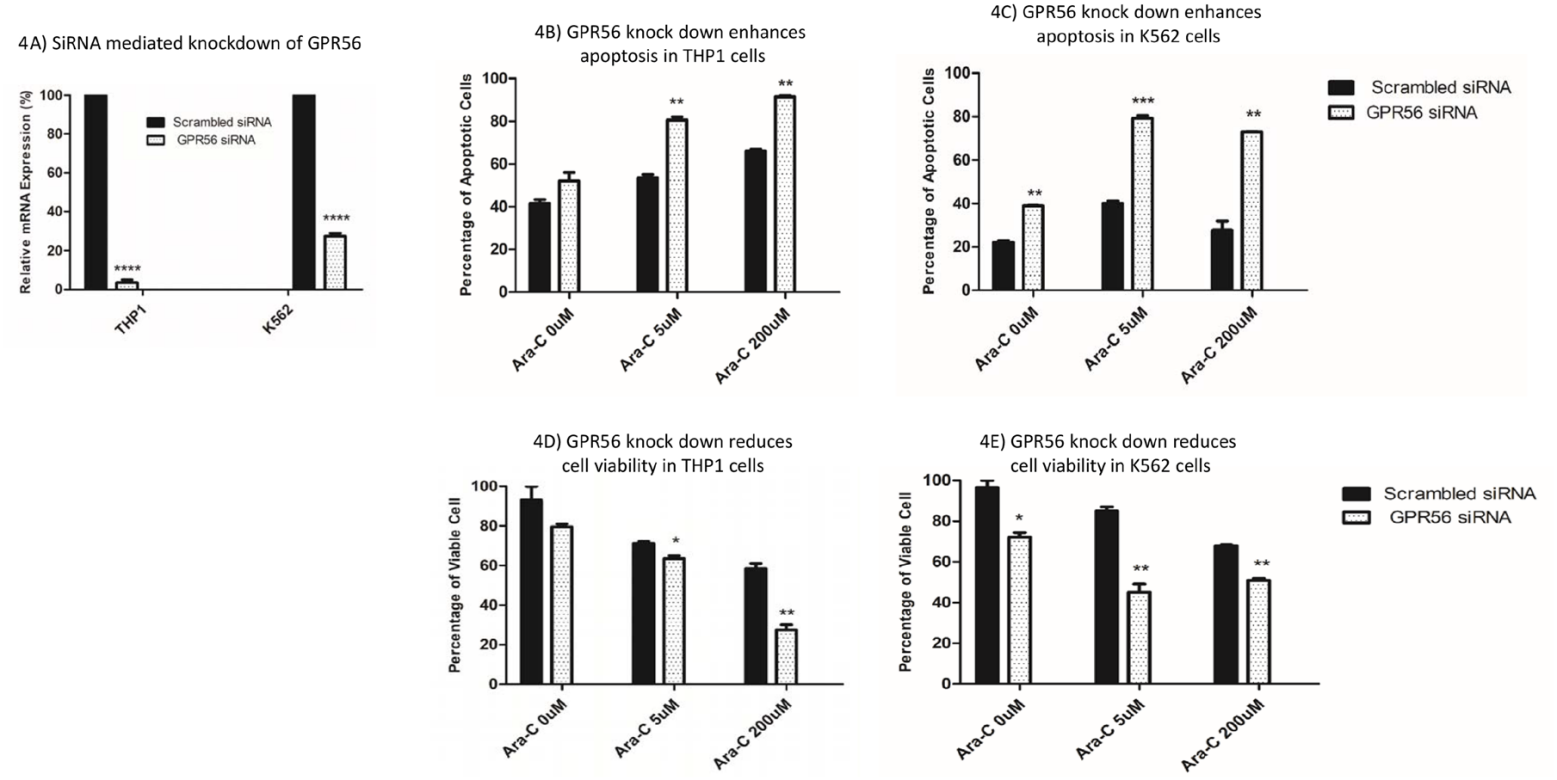
Impact of siRNA mediated knockdown of *GPR56* on cytarabine sensitivity in THP1 and K562 cell lines **(A)** *GPR56* mRNA levels in THP1 and K562 cells transfected with scrambled or GPR56 specific siRNAs. Impact of siRNA mediated knockdown of *GPR56* on apoptosis **(B** and **C)** and cell viability **(D** and **E)** post cytarabine treatment in THP1 and K562 cell lines. ^*^P<0.05, ^**^P<0.01, ^***^P<0.001, ^****^P<0.0001.

*GPR56* has been reported previously to play a role in leukemogenesis [20]and has been recently identified to be part of a 17-gene leukemia stem cell signature, LSC17.[29] The *GPR56* SNP rs1376041 G>A minor allele A was associated with cytarabine resistance (P=1.42x10^-5^, Figure 3A); greater *GPR56* mRNA expression levels in AML patients’ diagnostic leukemic cells were associated with resistance to cytarabine (P=0.036, Figure 3B) and consistent with these, presence of rs1376041 minor allele A was associated with greater *GPR56* mRNA expression in leukemic cells (P=0.022, Figure 3C). Other genes in the flanking region within 500kb of the *GPR56* SNP include other G protein-coupled receptors, namely *GPR97* and *GPR114* (Figure 3D). In addition to the *cise*QTL association of rs1376041 with *GPR56* expression, a significant association was also observed with the neighboring gene *KATNB1* (Table 3). We further investigated the impact of siRNA mediated transient knockdown of *GPR56* on cellular sensitivity to cytarabine. siRNA mediated knockdown resulted in a significant reduction in *GPR56* mRNA in both THP1 and K562 cell lines (P<0.0005, Figure 4A). GPR56 knockdown resulted in significant increase in apoptosis as shown by increase in Annexin V assay (P<0.01, Figure 4B and 4C) and reduction in cell viability (P<0.05, Figure 4D and 4E) in response to cytarabine at 5μM and 200μM of cytarabine in both the cell lines.

### rs75400242-*IGF1R*

**Figure 5:**
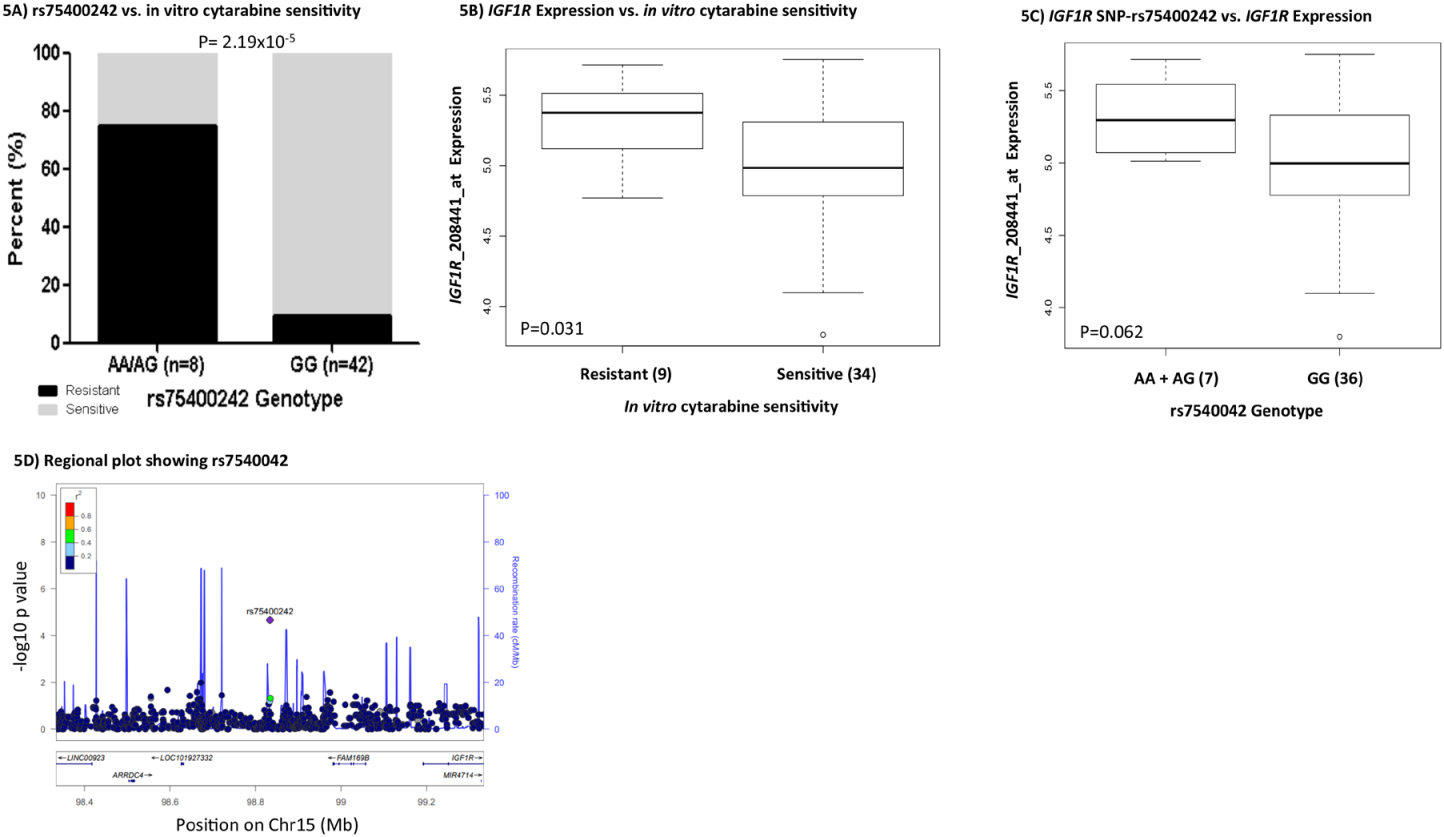
*IGF1R* and rs75400242 **(A)** Bar-plot showing distribution of cytarabine sensitive and resistant cases within genotype groups for rs75400242. **(B)** Boxplot showing association of *IGF1R* gene expression with cytarabine sensitive and resistant cases. **(C)** Boxplot demonstrating association of rs75400242 with *IGF1R* gene expression. In boxplots, the y-axis represents natural logarithm of gene expression and the x-axis shows the sensitive/resistant groups or genotype groups. The horizontal line inside the box represents the median value of each group, the lower hinge of the box represents the 25th percentile, the upper hinge represents the 75th percentile and the lower and upper whiskers here display 1.5 times the interquartile range. The outliers are defined as data points that fall outside of the first and third quartiles by more than 1.5 times the interquartile range. **(D)** showing -log10 p-value of the association of SNPs with cytarabine *in vitro* sensitivity on the y-axis and genes on chromosome 15 within +/- 500kb from this rs75400242 on the x-axis. As denoted by the legend with colors indicating linkage disequilibrium, rs75400242 was not in linkage disequilibrium with other SNPs within the region.

The second SNP-gene pair of interest is rs74500242- *IGF1R*, an insulin-like growth factor 1 receptor with tyrosine kinase activity. As shown in Figure 5A, the presence of the minor allele A for rs75400242 was associated with cytarabine resistance (P=2.19x10^-5^). Furthermore, *IGF1R* mRNA expression level within diagnostic leukemic cells was significantly higher in cytarabine resistant cases (P=0.031, Figure 5B). The presence of an A allele for rs75400242 showed a marginally significant association with greater *IGF1R* expression (P=0.062, Figure 5C). Other genes near rs75400242 include *ARRDC4* and *FAM169B* (Figure 5D).

**Figure 6:**
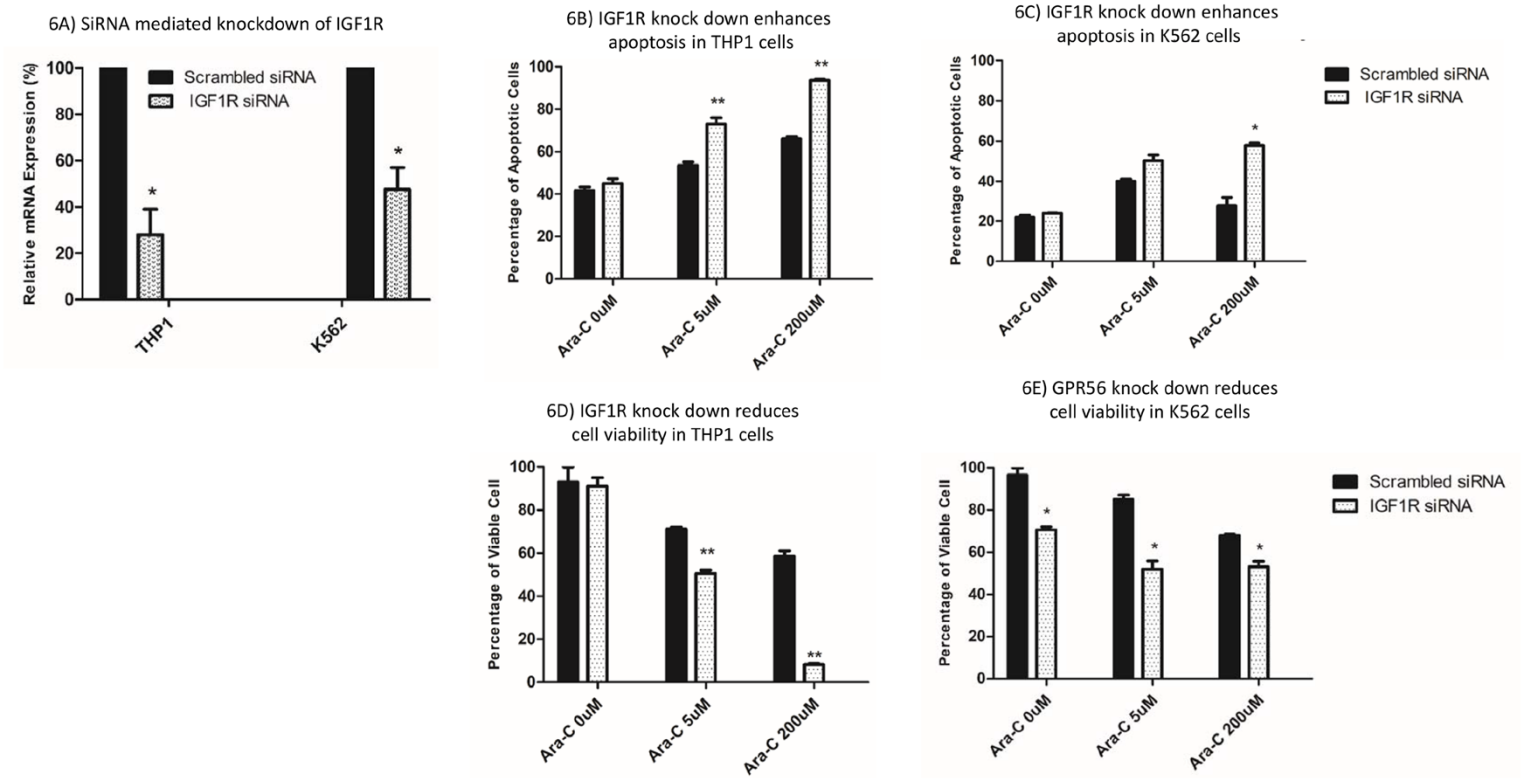
Impact of siRNA mediated knockdown of IGF1R on cytarabine sensitivity in THP1 and K562 cell lines **(A)** *IGF1R* mRNA levels in THP1 and K562 cells transfected with scrambled or *IGF1R* specific siRNAs. Impact of siRNA mediated knockdown of *IGF1R* on apoptosis **(B** and **C)** and cell viability **(D** and **E)** post cytarabine treatment in THP1 and K562 cell lines. ^*^P<0.05, ^**^P<0.01, ^***^P<0.001, ^****P<0.0001.^

We further investigated the impact of siRNA mediated transient knockdown of *IGF1R* on cellular sensitivity to cytarabine. siRNA mediated knockdown resulted in reduction in *IGF1R* mRNA in THP1 and K562 cell lines (P<0.05, Figure 6A). *IGF1R* knockdown enhanced apoptosis and correspondingly had reduced cell viability in response to cytarabine treatment, in THP1 cell lines (Figure 6B and 6D) and K562 cell lines (Figure 6C and 6E).

## DISCUSSION

**Figure 7:**
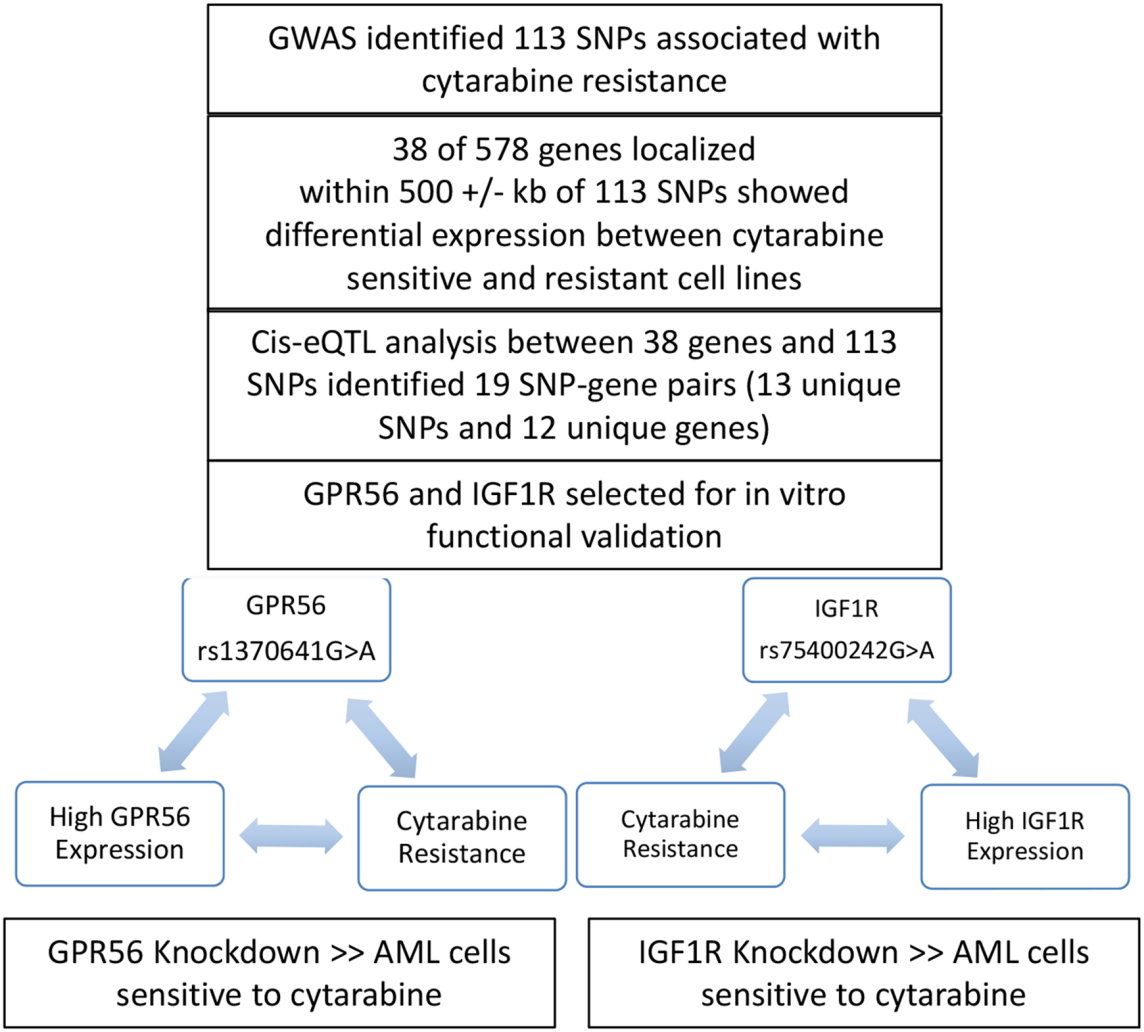
Overall summary of results from the current analysis

Development of resistance to cytarabine adversely impacts treatment outcome, making it very critical to understand the molecular mechanisms underlying cytarabine resistance. Past efforts to understand factors influencing cytarabine chemosensitivity have focused on the use of lymphoblast cell lines (LCLs) derived from healthy population as a model system or comparing gene-expression differences between parental and cytarabine resistant AML cells lines.[9–12] However, each of these systems have limitations as LCLs do not represent leukemic cells and AML cell lines can undergo changes in culture that might impact gene expression. In this study, we report the first GWAS analysis to identify genomic markers predictive of AML patients’ leukemic cell cellular sensitivity to cytarabine. We identified 113 SNPs associated with cytarabine *in vitro* sensitivity of leukemic cells obtained from pediatric AML patients. Within 500 kb of these SNPs, there were 38 genes that were differentially expressed between cytarabine sensitive and resistant cases. Nineteen SNP-gene pairs fulfilled the following three criteria: (1) association of SNPs with cytarabine *in vitro* sensitivity (p< 0.0001), (2) association of gene expression with cytarabine *in vitro* sensitivity (p<0.05) and (3) association of SNP with gene expression in a consistent direction (p < 0.1). Although this approach highlights significance of some SNPs, it is possible that other significant SNPs impact gene function by mechanisms other than regulating gene expression. Figure 7 summarizes the overall results of the current study. Although in-depth mechanistic investigation of all the 113 SNPs is beyond the scope of this work study, we selected two genes GPR56 and IGF1R for further *in vitro* validation. Consistent with the SNP and gene-expression results, siRNA mediated knockdown of these genes increased cellular susceptibility to cytarabine. *GPR56,* also commonly referred to as *ADGRG1*, encodes for G protein-coupled adhesion molecule and is highly expressed in leukemic stem cells and has been implicated in development of AML.[30] We have previously shown higher expression of GPR56 to be predictive of inferior outcome and more recently our data shows a significant relationship between GPR56 methylation and expression with outcome in pediatric AML.[18], Consistent with our findings, another study investigating DNMT3B as a player in leukemogenesis reported GPR56 as one of the target genes of DNMT3B and in adult AML high expression of GPR56 was associated with inferior overall survival. [31] GPR56 is also part of the 17 gene leukemic stem cell score (LSC17) that has been recently shown to have prognostic significance in predicting therapy resistance and relapse risk.[32] Saito *et al.* showed greater *GPR56* expression in AML samples with high ecotopic viral integration site-1 expression (EVI1^high^), which is considered a refractory type with poor prognosis. Further, *GPR56* knockdown in mice has been shown to result in an increase in cell migration and a decrease in cellular adhesion to the extracellular matrix, resulting in a reduction in cell growth rate and enhanced apoptosis.[20].

Our results show that the A allele of the *GPR56* rs1376041 SNP was associated with cytarabine resistance as well as with higher mRNA expression levels of *GPR56* gene, which in turn was associated with greater cytarabine resistance. This result indicates that rs1376041 SNP’s contribution to cytarabine *in vitro* sensitivity might actually be through its regulation of *GPR56* gene expression levels. This adds a unique level of evidence indicating *GPR56* rs1376041 SNP to be predictive of its expression and cytarabine chemosensitivity of patient leukemia cells. Further, *GPR56* knockdown in AML cell lines increased cytarabine sensitivity, suggesting its potential role in drug resistance and thus inferior outcome. To the best of our knowledge, even though recent data suggests significant role of GPR56 in AML, SNPs in GPR56 have not been studied in context of developing AML or resistance to treatment response.

The second signal, the rs75400242 SNP, is a G>A intronic SNP located 360 kb upstream of *IGF1R*, a gene encoding a transmembrane receptor protein having tyrosine kinase activity.[33, 34] Upon activation by insulin-like growth factor 1 (IGF-1), the IGF system regulates proliferation and differentiation of hematopoietic cells.[35] Activated *IGF1R*, can lead to upregulation of phosphoinositide 3 kinase/protein kinase B (PI3K/Akt) signaling, promoting growth and survival of AML cells.[23, 36] Cell growth and survival through *IGF1R* receptor-mediated activation of the PI3K/Akt signaling pathway has been reported previously [23, 37, 38], however its association with development of drug resistance is limited. Our results show *IGF1R* to be associated with cytarabine *in vitro* chemosensitivity and in conjunction with reports of specific targeting of IGF1/IGF1R signaling pathway having potent anti-leukemic activity in AML cells with constitutive PI3K activation, makes it a likely drug target.

Our study clearly adds another level of evidence by mapping a genetic variation that is predictive of leukemic cell cytarabine resistance. Although further studies are warranted to both validate the associations of SNPs identified in our study and to understand the functional underpinnings of the mechanisms contributing to cytarabine resistance, this is difficult due to the lack of such data sets owing to the practical challenges associated with obtaining *in vitro* drug sensitivity data from primary leukemia cells. To the best of our knowledge, even though limited by the relatively small sample size as compared to other GWAS studies, our study is the largest data set with *in vitro* cytarabine chemosensitivity in leukemic cells from pediatric AML patients.

In conclusion, although future work in larger clinical cohorts is warranted, our results identified SNPs, mapping to genes of relevance in AML.

## MATERIALS AND METHODS

### Patient cohort

This study included patients that were treated under the multicenter AML02 clinical trial (ClinicalTrials.gov identifier: NCT00136084). AML02 patients with t[8;21], inv [16], or t[9;11] chromosome abnormalities were classified as having low-risk AML. High-risk AML classification included those with -7, *FLT3*-ITD mutation, t[6;9], megakaryoblastic AML, treatment-related AML, or AML arising from MDS. Patients lacking low or high-risk group features were classified as standard-risk AML. Details of study population and outcome data has been previously reported.[2] Primary bone marrow samples at diagnosis from 65 patients were available to perform *in vitro* cytotoxicity study. The mononuclear cells were isolated using Ficoll-Hypaque density-gradient centrifugation within 24 h. Cells were resuspended in modified RPMI-1640 medium supplemented with 20% fetal calf serum, penicillin (100 IU/mL), streptomycin (100 μg/mL) and Fungizone™ (Invitrogen; 0.125 μg/mL), as well as ITS medium supplement containing insulin (5 ng/mL), transferrin (5 μg/mL), and sodium selenite (5 ng/mL), as previously described.[39] In the event the blast count was <80%, samples were enriched to achieve more than 80% blasts using magnetic cell sorting (Miltenyi Biotech, Germany). The sensitivity of leukemic cells to cytarabine was determined *in vitro* using the 4-day 3-(4,5-dimethylthiazol-2-yl)-2,5-diphenyltetrazolium bromide (MTT) cytotoxicity assay.[18] The leukemic cells were exposed to six different concentrations of cytarabine (ranging from 0.002–2.5 ng/μL) or to culture media (without drug) in a 96-well plate. MTT assay was performed after 96 hrs of drug treatment to determine cytarabine IC50. Fifteen patient samples did not achieve IC50 even at highest drug concentration tested, which was 2.5 ng/μL. Therefore, IC50 values from the 65 samples were dichotomized into those less than 5 ng/μL, which represented the sensitive group (n=50 total, n=40 Caucasians), and those that did not achieve IC50, represented as a resistant group (n=15 total, n=10 Caucasians).

### Genotyping

The genomic DNA samples of the AML02 participants were genotyped using the Illumina Omni 2.5M and Exome Beadchip (Illumina, San Diego, CA, USA) at Hussman Institute for Human Genomics, University of Miami, Miami FL, USA. Genotype calling was performed using Illumina's Genome Studio software V2011.1 (Illumina, San Diego, CA, USA).

### Gene expression

The mRNA expression levels in diagnostic leukemic blasts from the patient samples were previously determined using the Affymetrix U133A microarray data.[40] All gene expression values were natural log transformed prior to data analysis.

### Quality control procedure

QC steps were performed to obtain a high quality dataset for use in statistical analysis. QC is crucial since the capability of a GWAS to reveal true associations and the utility of those GWAS results is dependent on the quality of the data with significant impact on downstream analyses and replication studies.[41]The initial dataset had 2,612,357 variants and 65 participants. Marker QC comprised of filtering out 21,442 low quality SNPs having call rate ≤95% and 578,851 monomorphic SNPs. Of the remaining SNPs, the non-mitochondrial/non-haploid SNPs with MAF>5% were the ones analyzed. Multi-step sample QC procedure included identification and removal of individuals with a large proportion of SNPs failing i.e samples with a call rate ≤95%, individuals with mismatch between genetic and reported sex, related or duplicated individuals and individuals with outlying heterozygosity rate.[41, 42] Sample QC also confirmed the individual’s identified race and if genetic race was not consistent with their reported race, it was reassigned. All the samples passed sample QC steps and none were excluded in the analysis. Details of the QC procedure can be found in the Supplementary Table 2. All QC steps were performed using PLINK 1.9.[43, 44].

### Statistical analysis

Given the phenotype of interest, namely cytarabine IC50, was not normally distributed (Supplementarty Figure 1), the patients were classified into two groups: cytarabine sensitive: leukemic cells with IC50 less than 5 ng/μL, (n= 40 Caucasian patients), and cytarabine resistant: leukemic cells that never achieved IC50 and thus were assigned IC50 of 5 ng/μL, (n= 10 Caucasian patients). We utilized Cochran-Armitage trend test to assess SNPs associated with cytarabine sensitive or resistant groups within our white patients.[43, 44] Genome-wide significance was set at an alpha level of 5x10^-8^ [45] and a suggestive level was set at 1x10^-4^ for follow-up with gene expression data. All GWAS was conducted using PLINK 1.9.[43, 44].

### Gene-expression analysis

Differential gene expression for genes within +/-500kb of the significant/suggestive GWAS SNPs was conducted using Wilcoxon test to detect genes differentially expressed between the sensitive and resistant groups. *cis* eQTL analysis was conducted on candidate SNP-gene pairs identified in the GWAS and differential gene expression analysis above using Wilcoxon and Kruskal-Wallis tests, depending on how many genotype groups are available for each SNP. These analyses were done using R statistical software version 3.4.0.[46].

### *In vitro* functional validation

*In vitro* validation of two genes identified in GWAS and validated in gene expression analysis was performed using AML cell lines: THP-1, K562 (ATCC, Manassas, VA, USA). The cell lines were maintained in RPMI-1640 (Invitrogen, USA) supplemented with 10% FBS (Invitrogen, USA) at 37°C in a humidified atmosphere containing 5% CO2. The cells were passaged every 2–3 days in order to maintain them in logarithmic growth phase. siRNAs targeting *GPR56* and *IGF1R* as well as scrambled siRNA were procured from Dharmacon (Accell SMARTpool, USA). Just before transfection, the cells were grown in RPMI-1640-medium free of antibiotics. Delivery of siRNA at a final concentration of 50 nM was performed using RNAi Max reagent (Invitrogen, USA) according to the manufacturer's instructions. To monitor the effect of siRNA on gene silencing, transfection (5 × 105 cells/well) was done in 6-well plates for 48 hours. RNA levels of target genes *GPR56* and *IGF1R* as well as house-keeping gene *GAPDH* was then measured by Taqman gene expression assays (ThermoFisher Scientific, USA). Transfected cells harvested and exposed to cytarabine at 5 μM and 200 μM final concentration for 48 hours. Cells were stained with acridine orange (AO) and propidium iodide (PI) for viability assays and fluorescein isothiocyanate-conjugated Annexin V for apoptosis assays followed by manufacturer’s procedure, (Cellometer Inc., Lawrence, MA, USA). Cell imaging was undertaken by the Cellometer Vision CBA and FCS Express-6 software. In order to measure threshold in the software, it was set to 0% to quantify total fluorescence of each counted cell from the captured images. Based on fluorescence intensities, the results were calculated and converted to FCS file for analysis in the De Novo Software.55.

## SUPPLEMENTARY MATERIALS FIGURE AND TABLES



## Abbreviations

AML: Acute Myeloid Leukemia
Ara-C: 1-β-Arabinofuranosylcytosine or cytarabine
CML: Chronic Mylogenous Leukemia
eQTL: Expression Quantitative Trait Loci
EVI1: Ecotropic Viral Integration Site-1
GPR56: G Protein-Coupled Receptor 56
GWAS: Genome-Wide Association Analysis Study
HOXA10: Homeobox A10
HWE: Hardy-Weinberg Equilibrium
IBS/IBD: Identity-By-State/Identity-By-Descent
IC50: Half Maximal (50%) Inhibitory Concentration
IGF1: Insulin-like Growth Factor 1
IGF1R: Insulin like Growth Factor 1 Receptor
LCLs: Lymphoblastoid Cell Lines
LD: Linkage Disequilibrium
LincRNA: Long Intergenic Non-Coding RNA
MAF: Minor Allele Frequency
MN1: Meningioma 1
MRD: Minimal Residual Disease
MTT: 4-day 3-(4,5-dimethylthiazol-2-yl)-2,5-diphenyltetrazolium bromide
OPCML: Opioid Binding Protein/Cell Adhesion Molecule Like
OS: Overall Survival
PC: Principal Component
PCA: Principal Component Analysis
PI3K/Akt: Phosphoinositide 3 Kinase/Protein Kinase B
QC: Quality Control
