# Supplementary material for: Genome-wide association analysis identifies SNPs predictive of in vitro leukemic cell sensitivity to cytarabine in pediatric AML
Source: Oncotarget. 2018 Oct 9;9(79):34859–75. doi: 10.18632/oncotarget.26163 (PMC6201857; doi:10.18632/oncotarget.26163)
Supplement: Supplementary file 1 [file oncotarget-09-34859-s001.pdf]

Genome-wide association analysis identifies SNPs predictive of *in vitro* leukemic cell sensitivity to cytarabine in pediatric AML

SUPPLEMENTARY MATERIALS

Supplementary Table 1: List of genes with differential levels of expression between leukemic cell cytarabine sensitive and resistant groups of AML patients

Gene Symbol	P-value	Probe ID	Gene description	Expression in cytarabine resistant vs. sensitive cases	Direction of association wit High Gene Expression
SDHC	0.0000	215088_s_at	Succinate dehydrogenase complex subunit C	Low	Sensitive
SCARF1	0.0005	206995_x_at	Scavenger receptor class F member 1	HIGH	Resistant
PDHA2	0.0035	214518_at	Pyruvate dehydrogenase E1 alpha 2 subunit	HIGH	Resistant
TGFA	0.0048	205016_at	Transforming growth factor alpha	Low	Sensitive
FBN1	0.0060	202766_s_at	Fibrillin 1	HIGH	Resistant
SIGMAR1	0.0060	214484_s_at	Sigma non-opioid intracellular receptor 1	Low	Sensitive
NEDD9	0.0073	202150_s_at	Neural precursor cell expressed, developmentally down-regulated 9	HIGH	Resistant
MED13L	0.0143	212208_at	Mediator complex subunit 13 like	HIGH	Resistant
GRIN2D	0.0157	207036_x_at	Glutamate ionotropic receptor N-methyl D-aspartate type subunit 2D	Low	Sensitive
PCDHGA1-A8, PCDHGB1-4 family	0.0172	210368_at	Protocadherin gamma subfamily	HIGH	Resistant
PCDHGA1-A11, PCDHGB1-7 and PCDHGC3 family	0.0188	211066_x_at	Protocadherin gamma subfamily	Low	Sensitive
TOMM40	0.0223	202264_s_at	Translocase of outer mitochondrial membrane 40	Low	Sensitive
PCDHGA1	0.0223	209079_x_at	Protocadherin gamma subfamily A, 1	Low	Sensitive
HUNK	0.0223	219535_at	Hormonally up-regulated Neu-associated kinase	HIGH	Resistant
ARHGAP26	0.0243	205068_s_at	Rho GTPase activating protein 26	HIGH	Resistant
NME5	0.0264	206197_at	NME/NM23 family member 5	Low	Sensitive
DTNB	0.0264	215295_at	Dystrobrevin beta	Low	Sensitive
MTNR1A	0.0264	221369_at	Melatonin receptor 1A	HIGH	Resistant
ACVR1B	0.0286	208219_at	Activin A receptor type 1B	HIGH	Resistant
COPS2	0.0311	202467_s_at	COP9 signalosome subunit 2	HIGH	Resistant

(*Continued*)

HTRA2	0.0311	203089_s_at	HtrA serine peptidase 2	Low	Sensitive
IGF1R	0.0311	208441_at	Insulin-like growth factor 1 receptor	HIGH	Resistant
HIC1	0.0311	208461_at	Hypermethylated in cancer 1	HIGH	Resistant
PCDH12	0.0311	219656_at	Protocadherin 12	HIGH	Resistant
YY1	0.0336	200047_s_at	YY1 transcription factor	Low	Sensitive
ATP2C2	0.0336	206043_s_at	ATPase secretory pathway Ca2+ transporting 2	HIGH	Resistant
NPY6R	0.0336	210444_at	Neuropeptide Y receptor Y6 (pseudogene)	Low	Sensitive
PCDHGA11	0.0336	211877_s_at	Protocadherin gamma subfamily A, 11	HIGH	Resistant
CDIP1	0.0336	218183_at	Cell death-inducing p53 target 1	Low	Sensitive
BIN2	0.0336	219191_s_at	Bridging integrator 2	Low	Sensitive
ADGRG1 (GPR56)	0.0364	212070_at	Adhesion G protein-coupled receptor G1	HIGH	Resistant
STOML2	0.0393	215416_s_at	Stomatin like 2	Low	Sensitive
RPA1	0.0425	201529_s_at	Replication protein A1	Low	Sensitive
NAPG	0.0425	210048_at	N-ethylmaleimide-sensitive factor attachment protein gamma	HIGH	Resistant
BANP	0.0425	219966_x_at	BTG3 associated nuclear protein	HIGH	Resistant
KATNB1	0.0458	203163_at	Katanin regulatory subunit B1	HIGH	Resistant
PVR	0.0458	214444_s_at	Poliovirus receptor	Low	Sensitive
GFRA3	0.0493	214479_at	GDNF family receptor alpha 3	HIGH	Resistant

Supplementary Table 2: Summary table of QC Steps

Step	Results
AML02 GWAS Starting Data Set	2,612,357 variants and 65 subjects
Remove SNPs with missing data rate >5%	21,442 variants removed
Remove samples with missing data rate >5%	No subject removed
Remove monomorphic SNPs (MAF=0)	578,851 variants removed
Sex Check	No subject removed
Total Analyzable	2,012,064 – 694,918 (MAF<5%)- 40 (mitochondrial/ haploid)= 1,317,106 variants & 65 subjects
Extra QC:	
Additional QC	217,965 variants removed due to missing genotype data
	4,121 variants removed due to Hardy-Weinberg exact test.
	811,017 variants removed due to minor allele threshold
LD pruning	Ignoring 422 chromosome 0 variants (i.e 422 removed)
	826,771 variants removed
Removing variants on sex chromosome	3,948 variants removed
Pairwise IBS/IBD	No subject removed
PCA	Global PCA: adjust for PCs 1 and 2
	PCA by Caucasian race: adjust for PCs 1 and 2
	PCA by Black race: adjust for PC 1 only
Heterozygosity Analysis	No subject removed
Total	147,820 variants and 65 subjects

QC: quality control; GWAS: genome-wide association study; SNPs: single nucleotide polymorphisms; MAF: minor allele frequency; LD: linkage disequilibrium; IBS/IBD: identity-by-state/identity-by-descent; PCA: principal component analysis; PCs: principal components.

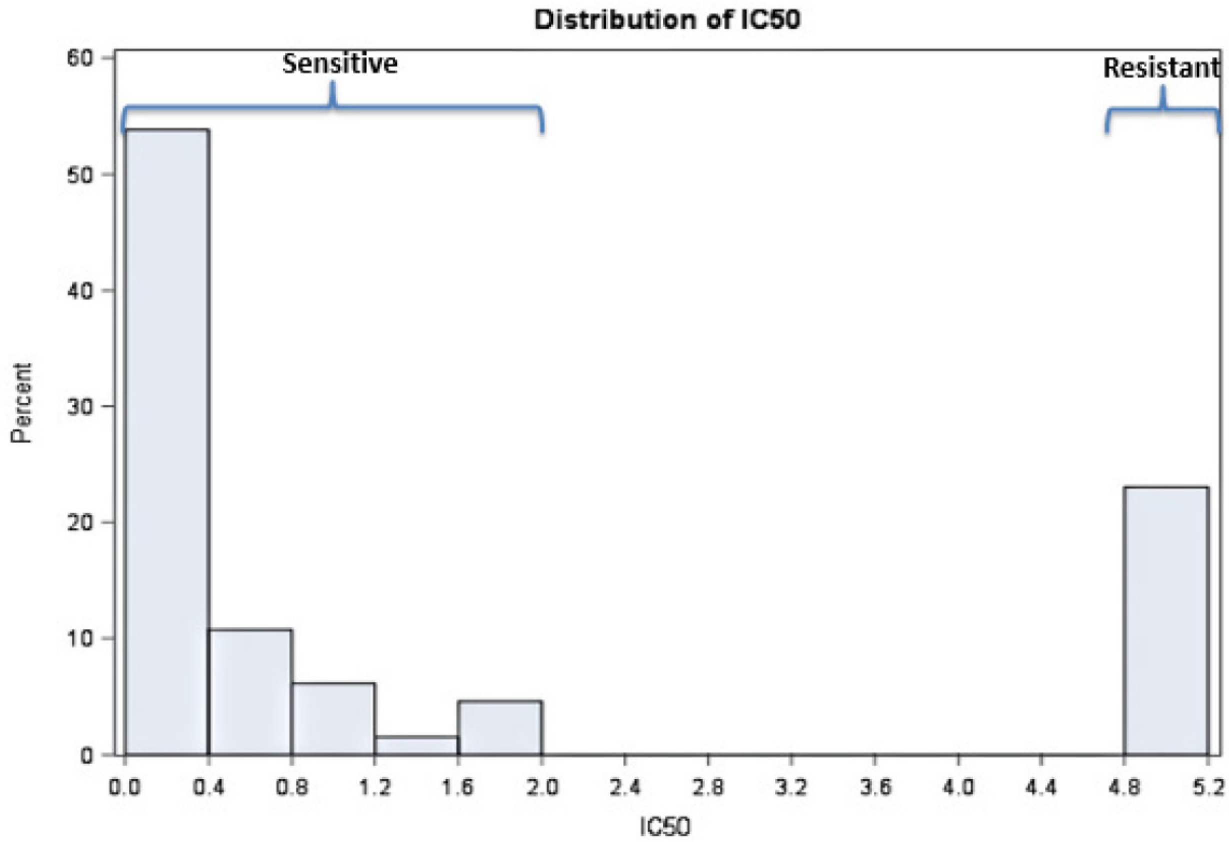

Supplementary Figure 1: Frequency distribution histogram showing distribution of cytarabine IC50 determined in leukemic cells obtained at diagnosis from pediatric AML patients (n=65). A natural gap in the data separates the sensitive group on the left who have an IC_{50} value < 5ng/μL and the resistant group on the right who never achieve IC_{50} in the concentration range tested and thus were designated an IC_{50} value of 5ng/μL (2x the highest tested concentration).
